# Ichthyosis Prematurity Syndrome Caused by a Novel Homozygous SLC27A4 Mutation in Two Emirati Siblings

**DOI:** 10.7759/cureus.96936

**Published:** 2025-11-15

**Authors:** Sara Almarzooqi, Fulvio Salvo

**Affiliations:** 1 Department of Dermatology, Sheikh Khalifa Medical City, Abu Dhabi, ARE; 2 Allergy and Clinical Immunology Division, Medical Specialty Institute, Cleveland Clinic Abu Dhabi, Abu Dhabi, ARE

**Keywords:** congenital ichthyosis, dupilumab, ichthyosis, ichthyosis congenita iv, ichthyosis prematurity syndrome, ips

## Abstract

Ichthyosis prematurity syndrome (IPS) is a rare autosomal recessive congenital ichthyosis caused by variants of the *SLC27A4* gene. It is characterized by the clinical triad of premature birth, clay-like vernix at birth, and respiratory complications. Although the skin manifestations tend to improve with age, patients may continue to exhibit mild ichthyosis and atopic manifestations. We describe the first cases of two Emirati siblings with IPS caused by a novel homozygous *SLC27A4* variant. Although the patients shared the same variant, they manifested differently, as one exhibited atopic features and the other suffered from recurrent infections. Interestingly, while both patients had elevated IgE levels and eosinophilia, only the patient with atopy responded to dupilumab.

## Introduction

Inherited ichthyoses constitute a large heterogeneous group of disorders of cornification that have traditionally been classified into syndromic and non-syndromic categories [1]. Recently, a novel classification has been proposed with the expanding understanding of the molecular basis of these disorders, and the terms syndromic and nonsyndromic epidermal differentiation disorders have been suggested to encompass ichthyoses, palmoplantar keratodermas, and other disorders of differentiation, to guide the consideration of new therapeutic targets [2]. Ichthyosis prematurity syndrome (IPS), also referred to as ichthyosis congenita type IV, is a rare disorder that belongs to the former group with a worldwide prevalence of 1 in 200,000 [3]. It is characterized by the triad of prematurity, clay-like vernix at birth, and respiratory complications [4]. While the ichthyosis usually improves with age, atopic diathesis (atopic dermatitis, asthma, and allergic rhinitis) may develop. IPS is caused by variants of the *SLC2A4* gene encoding the fatty acid transporter protein 4 (FATP4) [5] and is inherited in an autosomal recessive manner. Although IPS has a favorable prognosis, neonatal complications can be fatal without implementing respiratory support in cases of respiratory distress, and recognizing this entity is thus vital for appropriate and planned management.

## Case presentation

**Figure 1:**
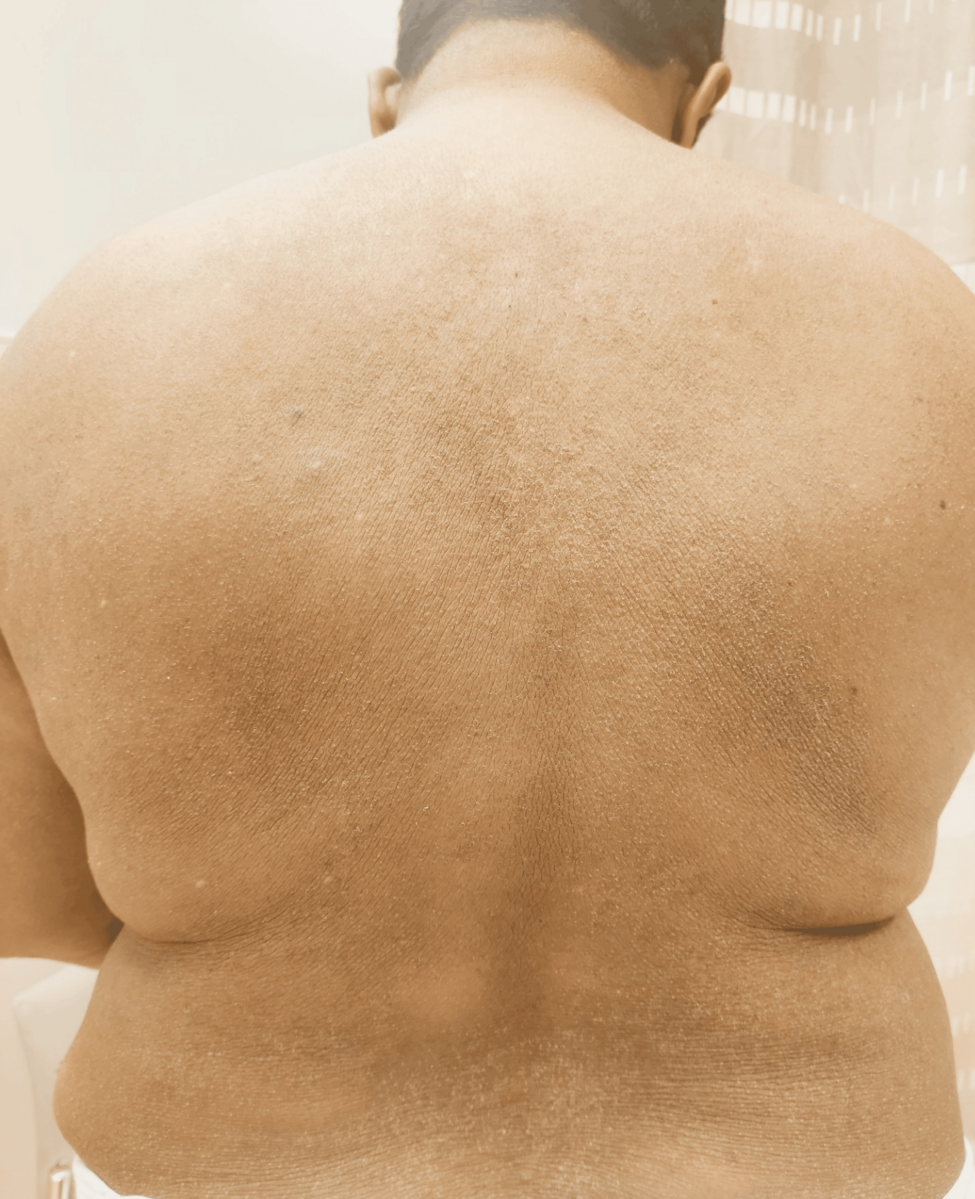
Accentuated skin markings of the central back, numerous scaly follicular papules

**Figure 2:**
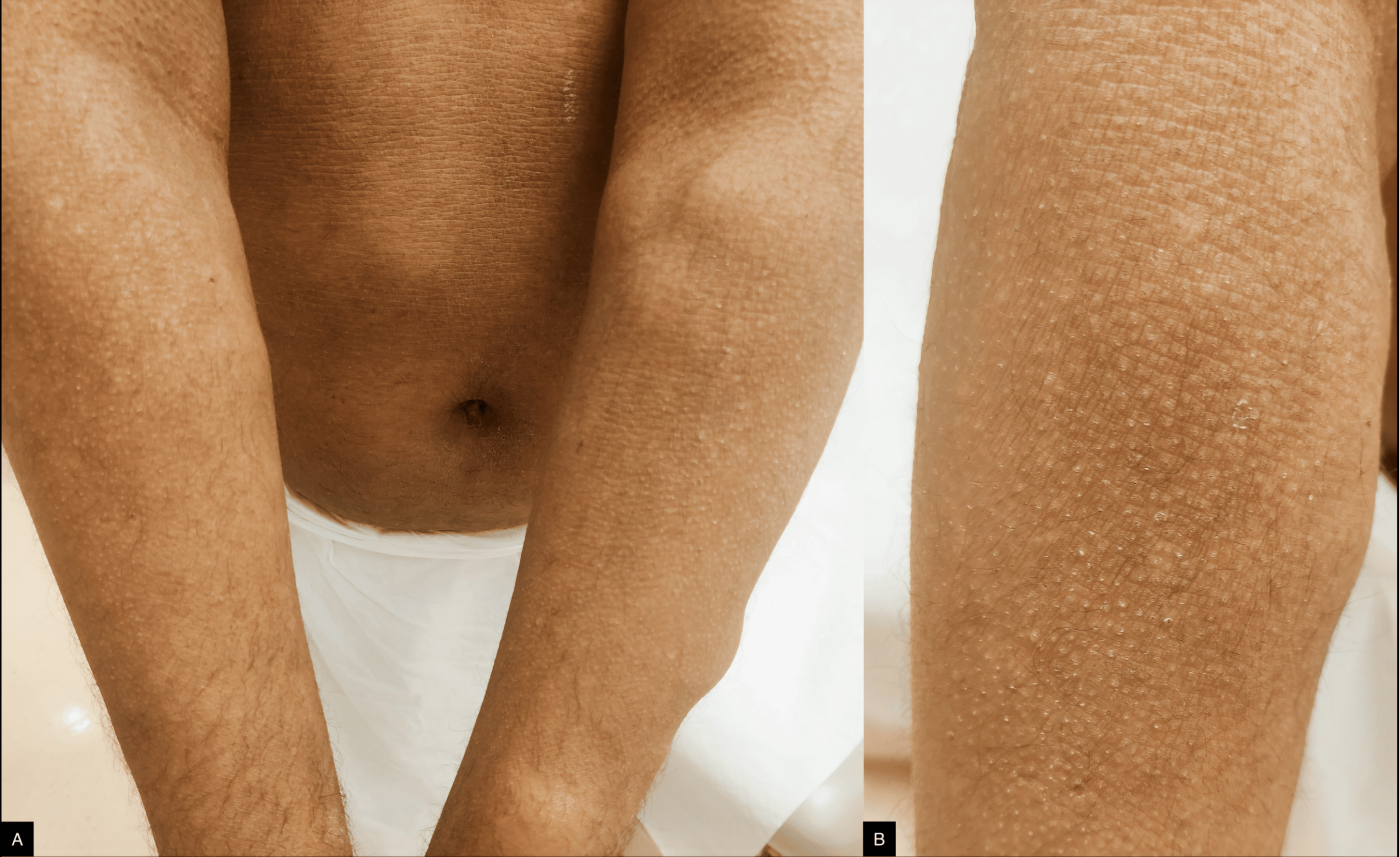
A. Scaly follicular papules on the extensor surface of the upper extremities. Accentuated skin markings on the abdomen. Note the multiple subcutaneous nodules (lipomas) on the left forearm. B: A close-up of the follicular papules.

Patient 1, a 43-year-old woman, presented with intense generalized pruritus impairing sleep, dry skin, and a likely diagnosis of congenital ichthyosis. This was associated with intermittent flares of inflammatory eczematous patches, mainly affecting the trunk and upper limbs. She also suffered from moderate asthma and was on inhaler therapy. On examination, she had generalized fine scales over her trunk and upper more than lower extremities, with relative sparing of the face, and accentuated flexural skin markings. Her laboratory work-up showed very high IgE levels of 19,770 IU/mL as determined by fluorescent enzyme immunoassay (normal range < 100 IU/mL, ImmunoCAP®, Thermo Fisher Scientific), and eosinophil count ranged from 4.46 to 6.77 x 10^9/L (normal range: < 0.70 x 10^9/L). Extensive workup, including bone marrow evaluation (cytogenetics, FIP1L1-PDGFRA RT-PCR, flow cytometry, T-cell clonality), parasitic studies, and serum tryptase, excluded myeloproliferative and lymphocytic hypereosinophilic syndrome, parasitic infections, and mastocytosis. The patient had asthma but no other features of eosinophilic granulomatosis with polyangiitis (EGPA) or specific organ involvement. Eosinophilia was present for many years and overall stable. A skin biopsy revealed moderate hypergranulosis and acanthosis, keratinocytes with basophilic appearance, and focal koilocytosis. In the dermis, a mild superficial perivascular lymphocytic infiltrate was noted, without eosinophilia. She had been using various topical agents, including a urea 10% cream and sedating antihistamines at night, with suboptimal response. She was commenced on dupilumab, 300 mg q2weeks. This led to significant improvement in pruritus and eczematous patches, and to a decrease in total IgE to 15,188 IU/mL, without further increase in the absolute eosinophil count. Her brother, Patient 2, is a 38-year-old man who first presented to the immunology/allergy clinic for frequent hospital admissions for cellulitis, chest wall abscess, and recurrent incision and drainage of abscesses in the groin and axillae; his last admission was due to a skin abscess caused by methicillin-resistant Staphylococcus aureus. He also had a history of generalized dryness, previously diagnosed as atopic dermatitis. He does not have a history of asthma or allergic rhinitis and denied any flares of eczematous patches. He was previously on dupilumab for six months with no benefit. His symptoms were controlled with emollients and antihistamines. Incidentally, he also had multiple lipomas on the trunk and extremities, similarly to his father, indicating a likely diagnosis of familial multiple lipomatosis. On examination, skin appeared hyperlinear with accentuated markings on the central back (Figure 1), abdomen, and upper extremities; and scaly follicular papules on bilateral extensor upper extremities (Figure 2) were noted. Laboratory investigations showed a serum IgE level of 33,244 IU/mL and an eosinophil count ranging from 1.76 to 2.84 × 10^9/L. Both patients were born prematurely at the 7th month of gestation to non-consanguineous, unaffected parents. They also have six unaffected siblings. Whole genome sequencing revealed a homozygous c.1645G>A p.(Gly549Arg) missense likely pathogenic variant in exon 12 (of 13) of the *SLC27A4* gene, which is predicted to have a deleterious effect on the gene or its product; this rendered the diagnosis of IPS likely. Variants linked to hyper IgE syndromes or other known inborn errors of immunity were not detected.

## Discussion

Ichthyosis prematurity syndrome (IPS; OMIM: 608649) has been widely characterized by the triad of premature birth, thick caseous desquamating epidermis at birth, and neonatal asphyxia [4]. Klar et al. first identified the underlying genetic variants in the *SLC27A4* gene, which encodes the FATP4, as the cause of IPS [5]. FATP4 is critical for skin barrier function during embryonic and neonatal periods, but its function may be compensated by other FATPs for maintenance, explaining the improvement of symptoms in patients with increasing age [5].

The majority of IPS cases have been reported in the Scandinavian population [6]. There are a few reported cases of IPS in the Middle East, a family described by Klar et al. [5], two Omani siblings [7], and a single case in Iraq [8] and Saudi Arabia [9]. To our knowledge, the described patients represent the first documented cases of IPS in the United Arab Emirates caused by a novel variant in the *SLC27A4* gene.

Prenatally, IPS findings on ultrasound include echogenic debris in the amniotic cavity and polyhydramnios, which may be caused by the obstruction of amniotic fluid flow through the gastrointestinal system due to the accumulation of epidermal debris in the fetus’s stomach [10]. Accumulation of this debris in the airways is also thought to be the cause of asphyxia shortly after birth [10]. This may necessitate ventilatory support and intensive care unit admission.

Patients present with thick, clay-like scales at birth, most commonly affecting the scalp, trunk, and upper extremities. Associated erythroderma is also present in most cases [3]. Often, skin manifestations resolve early. Cases describing adult patients with IPS are sparse; patients may have persistent mild ichthyosis, follicular hyperkeratosis, and atopic manifestations [3,6]. It is worth noting that the clinical phenotype can differ among patients sharing the same genetic mutation. In our case, one sibling exhibited more atopic features, including asthma and atopic dermatitis, whereas the other sibling experienced recurrent skin infections. Moreover, eosinophilia and very high IgE levels are common findings [3]. Multilamellar structures in the upper epidermis, demonstrated on electron microscopy, are a pathognomonic feature in IPS [3,4,6].

The successful use of dupilumab in other forms of congenital ichthyosis with atopic manifestations has been reported in the literature [11,12]. However, this was not reported previously in association with IPS, and as seen in our patients, response can vary even among individuals with the same genetic mutation.

Recognition of IPS is vital for genetic counselling, family planning, and reassuring parents of its benign course, aside from the potentially fatal risk of asphyxia perinatally [3,4]. This is also important for planning precautionary measures and appropriate management by the obstetrician and neonatologist.

## Conclusions

IPS is characterized by premature birth, thick scales at birth, and respiratory complications. Most IPS cases were reported in the Scandinavian population. Our patients represent the first reported cases in the United Arab Emirates, caused by a novel variant in the *SLC27A4* gene. Although our patients shared the same genetic variant, their clinical presentation and response to treatment differed. Recognizing this syndrome is crucial for genetic counseling and appropriate management in the neonatal period. While most cases of IPS tend to improve after early infancy, lifelong management may be required in a proportion of patients, not only through treatment of cutaneous symptoms, but also through early detection and management of possible associated comorbidities.
